# Assessing the Antioxidant, Hepatoprotective, and Iron-Chelating Potential of *Perilla frutescens* Seed

**DOI:** 10.3390/biomedicines13040851

**Published:** 2025-04-02

**Authors:** Sirichatnach Pakdeepromma, Komsak Pintha, Payungsak Tantipaiboonwong, Chonthida Thephinlap, Maitree Suttajit, Sawaruj Kaowinn, Napapan Kangwan, Wanwisa Suwannaloet, Kanjana Pangjit

**Affiliations:** 1 Department of General Science and Liberal Arts, King Mongkut’s Institute of Technology Ladkrabang Prince of Chumphon Campus, Chumphon 86160, Thailand; sirichat.ka@kmitl.ac.th; 2 Division of Biochemistry and Nutrition, School of Medical Sciences, University of Phayao, Phayao 56000, Thailand; komsakjo@gmail.com (K.P.); payungsak.t@gmail.com (P.T.); chonthida.th@up.ac.th (C.T.); maitree.suttajit@gmail.com (M.S.); 3 Department of Industrial Engineering, Faculty of Engineering, Chiang Mai University, Chiang Mai 50200, Thailand; sawaruj.k16@gmail.com; 4 Division of Physiology, School of Medical Sciences, University of Phayao, Phayao 56000, Thailand; napapan.ka@up.ac.th; 5 College of Medicine and Public Health, Ubon Ratchathani University, Ubon Ratchathani 34190, Thailand; wanwisa.su@ubu.ac.th

**Keywords:** *Perilla frutescens*, rosmarinic acid, iron chelation, antioxidant, hepatoprotective

## Abstract

**Background/Objectives:** Iron overload is a serious condition that can increase the production of reactive oxygen species (ROS), leading to oxidative tissue damage and organ dysfunction. While current pharmaceutical drugs for iron chelation have limitations, the search for natural herbs with iron-chelating properties is crucial. This study aimed to explore the various biological functions of the *Perilla frutescens* seed, regarding antioxidant activity and hepatoprotective and iron-chelating properties. **Methods:** *Perilla frutescens* seeds were subjected to extraction using a solvent-partitioning technique. Each fraction was evaluated for total phenolic content (TPC), total flavonoid content (TFC), and rosmarinic acid (RA) content by Folin–Ciocalteu assay, aluminum chloride colorimetric assay, and ultra-high-performance liquid chromatography (UHPLC), respectively. Antioxidant activity was assessed using DPPH, ABTS, and FRAP assays. The inhibition of lipid peroxidation was evaluated using the TBARS assay in HepG2 cells and an egg yolk model. The iron-chelating activity was examined using a ferric nitrilotriacetate (Fe^3+^-NTA)-binding assay, labile iron pool (LIP) level assessment, and the transferrin receptor (TfR) expression in HepG2 cells. **Results:** Phytochemical analysis indicated that the ethyl acetate (EtOAc) fraction had the highest TPC, TFC, and RA. This fraction demonstrated strong antioxidant properties and attenuated lipid peroxidation in HepG2 cells and egg yolk. In addition, this fraction exhibited iron-binding activity, decreased LIP levels, and induced TfR expression in iron-loaded HepG2 cells similar to the rosmarinic acid standard. **Conclusions:** These findings suggest that the EtOAc fraction of the *Perilla frutescens* seed possesses promising potential as a therapeutic agent for treating iron overload.

## 1. Introduction

Iron, an essential trace element for every living organism, plays major roles in many cellular processes such as energy metabolism, cell proliferation, regulation of gene expression, and many enzymatic reactions [1,2]. Ferrous ion is absorbed through divalent metal transporter 1 (DMT 1) in the duodenum and distributed throughout the body via the bloodstream, but there is no efficient mechanism for the excretion of excessive iron. Systemic iron homeostasis depends on the coordinated control of iron intake, transport, storage, and export [3]. The mechanism of cellular iron uptake involves the regulation of the transferrin receptor (TfR), a membrane transporter protein [4]. Excessive iron inhibits TfR expression, whereas low systemic iron increases TfR expression [5]. However, iron overload may occur when the body has more iron than it needs; excess iron can be deposited in the parenchymal cells of some organs and tissues, resulting in cell damage and organ dysfunction. The most direct effect of excess iron on cell damage is caused by the production of reactive oxygen species (ROS) such as hydroxyl radical (OH^•^) and superoxide anion (O_2_^•−^) via the Fenton and Haber–Weiss reactions [6,7]. ROS can cause lipid peroxidation by interacting with polyunsaturated fatty acids (PUFAs) in membranes, forming lipid free radicals (L^•^) and lipid reactive oxygen species (L-ROS). The accumulation of ROS can trigger oxidative stress, resulting in damage to DNA, proteins, and lipids in cells [8], which can lead to various diseases such as liver fibrosis, liver cancer [9], and hematological diseases [10]. Excessive intracellular iron is a labile iron pool (LIP) form, and it is the target for iron chelators to reduce ROS generation [11]. Three iron-chelating agents, deferoxamine, deferiprone, and deferasirox, are mainly used to treat iron overload. However, they have some disadvantages, such as their narrow scope of application, obvious side effects, and high prices [12]. Recent studies have demonstrated that phytochemical compounds from plants, such as polyphenols, flavonoids, and some vitamins, show a strong antioxidant activity that can block both lipid peroxidation [13,14] and iron overload [15,16]. Therefore, looking for effective phytochemical compounds that can serve as non-toxic antioxidants and inexpensive iron chelators is interesting.

*Perilla frutescens,* known as Nga-mon in Thai, has been extensively used in Southeast Asia for over a century as a common culinary, indigenous food ingredient and medicinal herb [17]. The different parts of this plant, including the leaves, seeds, and seed residue, have long been recognized for their beneficial active ingredients [18]. The phytochemicals present in *Perilla* contribute to its anti-inflammation, anti-cancer, anti-allergic, and antidepressant ability [19]. It has been reported that rosmarinic acid (RA) is the most prevalent phenolic compound in all regions of *Perilla* [20,21]. Previous studies found that the ethanolic extract of *Perilla* leaf, which mostly included rosmarinic acid as the major ingredient, showed antioxidant, iron-chelating, and anti-hemolytic properties [22]. However, no extensive studies have been carried out to investigate the effects of the RA-enriched fraction from *Perilla* seed on iron-chelating and anti-lipid peroxidation characteristics. Therefore, this study aimed to explore the protective effects of the RA-enriched fraction from *Perilla* seed against free radical production and for iron-chelating and anti-lipid peroxidation in human hepatoma cell lines (HepG2) and egg yolks.

## 2. Materials and Methods

### 2.1. Preparation of Rosmarinic Acid-Enriched Fraction from Perilla frutescens Seeds

*Perilla* seeds (PSs) cultivated in the Phayao region of Thailand were used in this investigation. A voucher specimen of the plant (code: QBG-93756) was gathered by W. Chaiwangyen and is stored at the Queen Sirikit Botanic Garden Herbarium in Chiang Mai, Thailand. The extraction of PS was performed using the method of Tantipaiboonwong and colleagues [21]. The *Perilla* seeds were cleaned, dried, and ground into a fine powder. This was then extracted with 70% ethanol with continuous stirring for 12 h. The extraction was carried out twice in the same condition. The supernatant was separated from the solid residue through Whatman No.1 filter paper. The filtrate was then evaporated in a rotary evaporator (BUSHI, Switzerland), followed by freeze-drying to obtain a crude ethanolic extract of *Perilla* seeds. Subsequently, 20 g of crude extract was redissolved in a 1:1 hexane–water mixture and purified by solvent extraction with hexane (Hex), dichloromethane (DCM), and ethyl acetate (EtOAc), respectively. Each fraction was dried under reduced pressure to obtain a powder and was stored at −20 °C until use.

### 2.2. Determination of Rosmarinic Acid Content in Perilla Seed Extract

The rosmarinic acid (RA) in *Perilla* seed extract was analyzed using the ultra-high performance liquid chromatography (UHPLC) system. Each fraction of *Perilla* seed extract was filtered and injected on the UHPLC system (Agilent Technologies, Inc., Santa Clara, CA, USA) containing a C18 column (150 mm × 4.6 mm × 5 μm). The UHPLC chromatogram of RA was determined and compared with the RA standard. The mobile phase contained a mixture of 0.1% trifluoroacetic acid and acetonitrile. The flow rate was set at 1.0 mL/min, and the detection of RA was measured at 280 nm [23,24].

### 2.3. Determination of Total Phenolic Content

Total phenolic content (TPC) was determined using the Folin–Ciocalteu assay [25]. In brief, 20 μL of each fraction was combined with 80 μL of 7.5% sodium carbonate and 100 μL of 10% Folin–Ciocalteu reagent. The mixture was kept in the dark at room temperature for 15 min. The absorbance was measured using a microplate reader (Metertech; Taiwan) at 750 nm. Total phenolic content was expressed as gallic acid equivalents in milligram per gram of extract (mg GAE/g extract).

### 2.4. Determination of Total Flavonoid Content

Total flavonoid content (TFC) was determined by the aluminum colorimetric method assay [25]. The 25 µL of each fraction was mixed with 7.5 μL of 5% NaNO_2_ in the dark for 6 min. Then, 15 µL of 10% AlCl_3_ and 50 μL of 1.0 M NaOH was added to the mixture, which was kept at room temperature for 10 min. The absorbance of the solution was analyzed at 510 nm using a microplate reader (Metertech; Taiwan). Total flavonoid content was expressed as catechin equivalents in milligrams per gram of extract (mg CE/g extract).

### 2.5. DPPH Radical Scavenging Assay

The DPPH radical scavenging activity was determined according to the method of Tipsuwan and Chaiwangyen, 2018, with slight modifications [22]. In a 96-well plate, an aliquot (180 µL) of 0.2 mM DPPH reagent was mixed with varying concentrations of the fractioned *Perilla* seed extract (20 µL). After a 15 min incubation period in the dark at room temperature, the absorbance of the mixture was measured at 540 nm using a microplate reader (Metertech; Taiwan). Each sample was analyzed in triplicate for DPPH scavenging activity, compared to standard vitamin C and trolox. Antioxidant activity is expressed as the extract concentration that scavenged 50% of free radicals (IC_50_), calculated from the graph of %DPPH scavenging activity against fractioned *Perilla* seed extract concentration. The percentage of DPPH radical scavenging activity was determined as follows:

$$\%\mathrm{DPPH}\;\mathrm{radical}\;\mathrm{scavenging}\;\mathrm{activity}\;=\frac{\left({\mathrm{Abs}}_{\left(\mathrm{control}\right)}-{\mathrm{Abs}}_{\left(\mathrm{sample}\;\mathrm{or}\;\mathrm{vit}\;C\;\mathrm{or}\;\mathrm{trolox}\right)}\right)\;}{{\mathrm{Abs}}_{\left(\mathrm{control}\right)}}\times 100$$

### 2.6. ABTS Radical Scavenging Assay

The ABTS radical scavenging activity was determined according to the method of Tipsuwan and Chaiwangyen, 2018, with slight modifications [22]. In a 96-well plate, an aliquot (190 µL) of ABTS solution was mixed with varying concentrations of the fractioned *Perilla* seed extract (10 µL). The solution mixture was incubated in the dark for 6 min at room temperature and then measured at 734 nm by a microplate reader (Metertech; Taiwan). Each sample was analyzed in triplicate. The antioxidant potential of the extract was assessed by measuring its ability to scavenge ABTS radicals compared to vitamin C and trolox. Antioxidant activity was expressed as the IC50 value, which represents the concentration needed to inhibit 50% of free radicals. This IC50 value was calculated by plotting the percentage of ABTS radical scavenging activity against varying concentrations of the fractionated *Perilla* seed extract. The percentage of ABTS radical scavenging activity was calculated as follows:

$$\%\mathrm{ABTS}\;\mathrm{scavenging}\;\mathrm{activity}\;=\frac{\left({\mathrm{Abs}}_{\left(\mathrm{control}\right)}-{\mathrm{Abs}}_{\left(\mathrm{sample}\;\mathrm{or}\;\mathrm{vit}\;C\;\mathrm{or}\;\mathrm{trolox}\right)}\right)\;}{{\mathrm{Abs}}_{\left(\mathrm{control}\right)}}\times 100$$

### 2.7. Ferric Reducing Antioxidant Power Assay

The ferric reducing antioxidant power of the extracts was determined as described by Benzie and Strain [26]. Briefly, a FRAP working solution was prepared using a 300 mM acetate buffer at pH 3.6, 40 mM solution of 2,4,6-tri(2-pyridyl)-s-triazine (TPTZ), and 20 mM ferric chloride in a 10:1:1 volume ratio. Subsequently, 900 µL of the prepared FRAP reagent was combined with 30 µL of the sample, with all measurements performed in triplicate. The reaction mixture was incubated for 30 min in the dark, and absorbance was measured at 593 nm. FeSO_4_ was used as a standard, and the results were expressed in mg ferrous ion (Fe^2+^) equivalents/g extract.

### 2.8. Cell Culture

The human hepatoma cell line (HepG2) and the murine macrophage cell line (RAW 264.7) used in this study were sourced from the American Type Culture Collection (ATCC, Manassas, VA, USA). These cells were grown in DMEM medium enriched with 10% fetal bovine serum (FBS) and 1% penicillin/streptomycin. The cells were maintained in a humidified incubator at 37 °C, with 5% carbon dioxide. Subculturing occurred when the cells reached approximately 80% confluence.

### 2.9. Cell Viability Assay

The cell viability assay was analyzed using an MTT assay [25]. In brief, the HepG2 cells (5 × 10^3^ cells/well), or RAW 264.7 cells (1 × 10^4^ cells/well) were plated into a 96-well plate for 24 h. Next, the cells were pretreated with various concentrations of rosmarinic acid (1.56–100 μg/mL) or EtOAc fraction (12.5–200 μg/mL) for 24 h. Then, 5 mg/mL MTT dye was added to the treated cells in each well, then incubated for 4 h at 37 °C and 5% CO_2_. All supernatants were removed, and DMSO was then added to the well. The absorbance was measured at 540 nm using a microplate reader (CLARIOstar^®^, BMG LABTECH, Ortenberg, Germany). All measurements were performed in triplicate.

### 2.10. Intracellular ROS Production

A 2′,7′dichlorodihydrofluorescein diacetate (DCFH-DA) assay was used to measure the accumulation of intracellular ROS in the HepG2 cells. The cells were seeded into 96-well black plates (5 × 10^3^ cells/well) and treated with various concentrations of rosmarinic acid, EtOAc fraction, or trolox for 24 h. After an incubation period, the cells were washed twice with phosphate-buffered saline (PBS) pH 7.4. The cells were incubated with 10 μM of DCFH-DA at 37 °C in 5% CO_2_ incubator for 30 min. Subsequently, the HepG2 cells were exposed to 125 μM H_2_O_2_ at 37 °C in a 5% CO_2_ incubator for 10 min. The fluorescence intensity was measured using a fluorescence microplate reader (CLARIOstar^®^, BMG LABTECH, Ortenberg, Germany) at excitation and emission wavelengths of 485 nm and 530 nm, respectively [27]. All measurements were performed in triplicate.

### 2.11. Determination of Lipid Peroxidation in HepG2 Cells

Lipid peroxidation was determined by the thiobarbituric acid reactive substances (TBARS) assay [28]. HepG2 cells were seeded into 6-well plates (5 × 10^6^ cells/well) and incubated with 1 mM ferrous ammonium sulfate (FAS) for 24 h. The cells were incubated with rosmarinic acid, EtOAc fraction, or trolox at various concentrations at 37 °C in 5% CO_2_ for 24 h. The cells were collected and lysed with ultrasonication. The cell lysate was mixed with 15% trichloroacetic acid (TCA) and 0.675% thiobarbituric acid (TBA) and then heated in a 90 °C water bath for 60 min. After cooling, the mixture solutions were centrifuged, and the absorbance of the supernatant was measured at 532 nm using a microplate reader (CLARIOstar^®^, BMG LABTECH, Ortenberg, Germany). The malondialdehyde (MDA), the end product of lipid peroxidation, was calculated by using 1,1,3,3-tetraethoxypropane as the standard. The results are expressed as nM/μg protein.

### 2.12. Inhibition of Lipid Peroxidation in Egg Yolk

The lipid peroxidation inhibition of rosmarinic acid and the EtOAc fraction in egg yolk was performed according to the previous method with some modifications [29]. In brief, 100 μL of EtOAc fraction (10–80 μg/mL), rosmarinic acid (5–40 μg/mL), or trolox (12.5–100 μg/mL) was added to 500 µL of egg yolk prepared in phosphate-buffered saline (PBS) at a ratio of 1:4 (*w*/*v*). Trichloroacetic acid and 400 µL of 0.8% thiobarbituric acid was added, followed by 100 µL of 20 mM ferrous sulfate (1 mM) and 900 µL of distilled water. After shaking, the mixture was boiled at 95 °C for 30 min; after cooling, the mixture was centrifuged at 6000 rpm for 5 min at 25 °C. The absorbance was measured at 532 nm by UV–VIS spectrophotometer (Thermo Scientific, GENESYS 180, Waltham, MA, USA). The malondialdehyde (MDA), the end product of lipid peroxidation, was calculated using 1,1,3,3-tetraethoxypropane as the standard. The results were expressed as µM of MDA.

### 2.13. Nitric Oxide Assay

The RAW 264.7 cells were plated into a 24-well plate (1 × 10^5^ cells/well) for 24 h. Next, the cells were incubated with LPS (2 µg/mL) for 24 h. The cells were incubated with different concentrations of the EtOAc fraction (10, 20, and 40 µg/mL) or rosmarinic acid (5, 10, and 20 µg/mL) for 24 h. N(gamma)-nitro-L-arginine methyl ester (L-NAME) (50 µg/mL) was used as the positive control. Afterward, the 100 µL of supernatant was incubated with Griess reagent at room temperature for 20 min. The reagent mixtures were measured at 540 nm against a blank reagent, using a microplate reader. The total nitrite concentration was calculated using the standard curve prepared with sodium nitrite and Griess reagent solutions.

### 2.14. Ferric (Fe^3+^) Ion Chelation Assay

The ion-chelating properties of the EtOAc fraction were determined by spectrophotometry with slight modifications [16]. Ferric nitrilotriacetate (Fe^3+^-NTA) solution (0–200 μM) was incubated with the EtOAc fraction (500 μg/mL), rosmarinic acid (100 μg/mL), or deferiprone (14 μg/mL) at a pH of 7.0 for 1 h. The ferric ion complexes were measured in the 220–900 nm wavelength range using a UV–VIS spectrophotometer (Thermo Scientific, GENESYS 180, Waltham, MA, USA). The EtOAc fraction (500 μg/mL) or rosmarinic acid (100 μg/mL) solution was used as a blank. The working Fe^3+^-NTA solution was prepared by mixing a stock standard iron solution with nitrilotriacetate (NTA) solution in 50 mM MOPS (3-(N-Morpholino) propanesulfonic acid, 4-Morpholinepropanesulfonic acid) solution, pH 7.0, with a molar ratio of ferric ion/NTA = 1:5.

### 2.15. Intracellular Iron Chelation Assay

The intracellular iron chelation assay of the EtOAc fraction was determined with the calcein acetoxymethyl ester (Calcein-AM) fluorescence technique [16]. The HepG2 cells were seeded into 96-well plates (5 × 10^3^ cells/well) and exposed to 0.4 mM ferric ammonium citrate (FAC) for 24 h. After 24 h incubation, the cells were treated with the EtOAc fraction or rosmarinic acid at various concentrations, then incubated at 37 °C in a 5% CO_2_ incubator for 24 h. The treated cells were incubated with 1 μM Calcein-AM (Invitrogen, Waltham, MA, USA) for 15 min, then washed with PBS, and the fluorescence intensity was measured at λ_ex_ 485 nm and λ_em_ 535 nm with a fluorescence microplate reader. LIP levels were inverse to the fluorescence intensity.

### 2.16. Transferrin Receptor (TfR) Expression Assessment

Transferrin receptor (TfR) expression on hepatocyte membranes implicated intracellular iron deprivation. Hepatic TfR expression was analyzed using RT-qPCR. HepG 2 cells (5 × 10^5^ cells) were plated in 24-well plates at 37 °C in a 5% CO_2_ incubator for 24 h. The cells were treated with 400 μM FAC for 24 h. The treated cells were incubated with the EtOAc fraction or rosmarinic acid at various concentrations for 24 h. Total RNA was extracted using the GF-1 RNA extraction kit (Vivantis, Selangor, Malaysia). The amount of RNA was quantified using a Nanodrop 2000 spectrophotometer (Thermofisher Scientific, USA). Total RNA being reverse-transcribed into cDNA was performed using iScript™ reverse transcription supermix (Bio-Rad, Hercules, CA, USA). The template cDNA was used for RT-qPCR amplification with LightCycler^®^ 480 SYBR Green I Master (Roche, Atlanta, GA, USA). The forward primer sequence of *TfR* was 5′-CTGCGGCTAGTGGTCAACAA-3′, while the reverse primer sequence was 5′-GCCGGAAGCCGTTGCT-3′. The forward primer of *gapdh* was 5′-TCATC AGCAATGCCTCCTGCA-3′, and the reverse primer was 5′-TGGGTGGCAGTGATGG CA-3′. The mRNA levels of *TfR* were normalized using *gapdh* as an internal control, and relative quantitation was calculated by the 2^−∆∆Ct^ method.

### 2.17. Statistical Analysis

Data were analyzed using the GraphPad Prism 9.0 software and were expressed as the means ± standard deviation (SD) of three independent experiments. Statistical significance was determined using a one-way analysis of variance (ANOVA), followed by Dunnett’s post hoc test, for which *p*-values < 0.05, 0.01, and 0.001 were considered significantly different.

## 3. Results

### 3.1. Extraction Yields, TPC, TFC, and RAC in PS Extract and Its Fractions

*Perilla* seed (PS) extracted with 70% ethanol (EtOH) yielded 18.68% (*w*/*w*). Then, the ethanolic extract of the PS was partitioned with hexane (Hex), dichloromethane (DCM), ethyl acetate (EtOAc), and water, respectively. The extraction yield, total phenolic content (TPC), total flavonoid content (TFC), and rosmarinic acid content (RAC) are presented in Table 1. The extraction yield was highest in the water fraction (70.40% *w*/*w*), followed by the Hex (5.79% *w*/*w*), DCM (2.69% *w*/*w*), and EtOAc (2.24% *w*/*w*) fractions. The TPC in the ethanolic extract was 180.56 ± 4.47 mg GAE/g extract. The TPC in the Hex, DCM, EtOAc, and water fractions were 80.60 ± 6.62, 192.70 ± 5.65, 1314.05 ± 23.04, and 136.12 ± 1.69 mg GAE/g extract, respectively. TFC in the ethanolic extract was 72.27 ± 3.58 mg CE/g extract. The TFC in the Hex, DCM, EtOAc, and water fractions were 17.96 ± 0.73, 35.26 ± 1.35, 329.30 ± 10.37, and 62.66 ± 2.47 mg CE/g extract, respectively.

**Figure 1:**
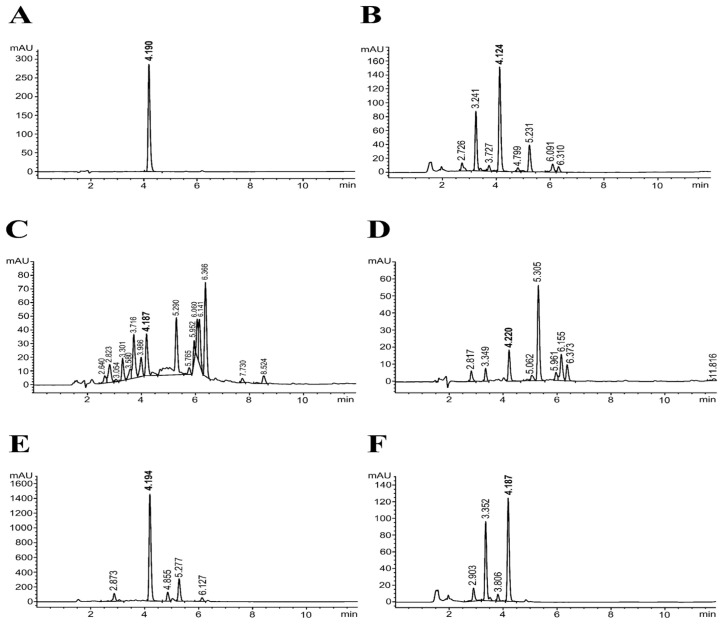
Ultra-high performance liquid chromatogram obtained from the analysis of rosmarinic acid (RA) standard (**A**), ethanolic extract (EtOH) (**B**), and solvent-partitioning extract fraction: hexane (Hex) (**C**), dichloromethane (DCM) (**D**), ethyl acetate (EtOAc) (**E**), and water (**F**).

The UHPLC chromatogram of the rosmarinic acid (RA) standard showed the major peak at a retention time (RT) of 4.190 min (Figure 1A). The quantification of RA content in each fraction was achieved using the standard curve, which found that the RA content in 70% ethanolic extract was 41.77 ± 0.02 mg/g extract. The RA content in the Hex, DCM, EtOAc, and water fractions was 4.74 ± 0.20, 10.42 ± 0.07, 393.81 ± 0.05, and 32.92 ± 0.01 mg/g extract, respectively. Based on the data presented in Table 1 and Figure 1, the EtOAc fraction is observed to have the highest content of TPC, TFC, and RA. According to these results, the EtOAc fraction was selected for further characterization of its antioxidant activity against oxidative stress and lipid peroxidation and its iron-chelating properties.

### 3.2. Antioxidant Abilities and Reducing Power Property of PS Extract and Its Fractions

The discovery of antioxidant properties in PS extract and its fractions using DPPH, ABTS, and FRAP assay is addressed in this part. An examination of Table 1 reveals the ability to scavenge free radicals measured by the DPPH and ABTS techniques. The EtOAc fraction showed the highest antioxidant activity, with the lowest IC_50_ values of 13.35 ± 0.81 µg/mL compared to the other extract fractions measured by DPPH. Comparison with rosmarinic acid and reference antioxidants (the IC_50_ values of rosmarinic acid, vitamin C, and trolox were 8.69 ± 0.37, 5.47 ± 0.11, and 5.20 ± 0.02 µg/mL, respectively) indicated that the EtOAc fraction exhibited antioxidant activity. Likewise, the IC_50_ of ABTS scavenging activity of the EtOAc fraction was 3.98 ± 0.13, compared with rosmarinic acid and reference antioxidants (the IC_50_ values of rosmarinic acid, vitamin C, and trolox were 5.45 ± 0.04, 5.18 ± 0.06, and 5.17 ± 0.04 µg/mL, respectively). Thus, the EtOAc fraction plays an important role in free radical scavenging.

The FRAP assay was used to analyze the antioxidant properties of the PS extract and its fractions; the results were expressed as FRAP values (mg Fe^2+^ equivalent/g extract) (Table 1). The basis of the FRAP assay is the antioxidant’s ability to reduce ferric ion (Fe^3+^) to ferrous ion (Fe^2+^) in the presence of TPTZ (2,4,6-tris(2-pyridyl)-s-triazine), which forms a blue Fe^2+^-TPTZ complex with a maximum absorbance at 593 nm. Higher FRAP values indicate a higher reducing power. Our results showed that the EtOAc fraction demonstrated the highest reducing power capacity (5062.95 ± 354.87 mg Fe^2+^ equivalent/g extract) compared to the other extract fractions. It was observed that the EtOAc fraction exhibited strong reducing powers, similar to those of standard rosmarinic acid, vitamin C, and trolox (7775.37 ± 439.56, 4347.10 ± 57.09, and 4885.00 ± 95.35 mg Fe^2+^ equivalent/g extract, respectively). According to these findings, the EtOAc fraction exhibited potential free radical scavenging and reducing power properties in vitro.

### 3.3. Cytotoxic Effect of EtOAc Fraction

**Figure 2:**
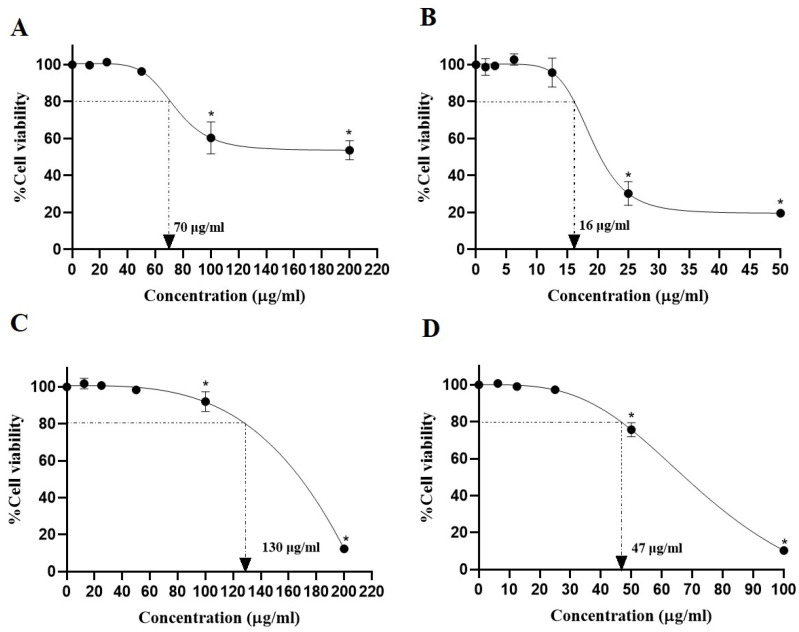
The cytotoxic effect of the EtOAc fraction (**A**) and rosmarinic acid (**B**) on HepG2 cells and the cytotoxic effect of the EtOAc fraction (**C**) and rosmarinic acid (**D**) on RAW 264.7 cells at 24 h of incubation. All assays were performed in triplicate; data are presented as mean ± standard deviation (* *p* < 0.05 vs. the control group).

The toxicity of the EtOAc fraction was studied by MTT assay on human hepatoma cells (HepG2) and murine macrophage RAW 264.7 cells. The percentage of surviving cells was examined, following a 24 h exposure. As demonstrated in Figure 2, concentrations less than 70 µg/mL of the EtOAc fraction exerted no cytotoxic effects in both HepG2 and RAW 264.7 cells (IC_20_ values were 70 and 130 µg/mL, respectively). Subsequently, the non-toxic dose of rosmarinic acid on HepG2 and RAW 264.7 cells was evaluated, showing IC_20_ values of 16 and 47 µg/mL, respectively. No significant cytotoxicity was observed in HepG2 cells treated with trolox (10–40 µg/mL) or deferiprone (20 µg/mL) or in RAW 264.7 cells treated with N(gamma)-nitro-L-arginine methyl ester (L-NAME) (50 µg/mL) which was used as a positive control. Therefore, the non-toxic concentrations were used for all of the following experiments.

### 3.4. Effect of EtOAc Fraction on Intracellular ROS Generation in HepG2 Cells

**Figure 3:**
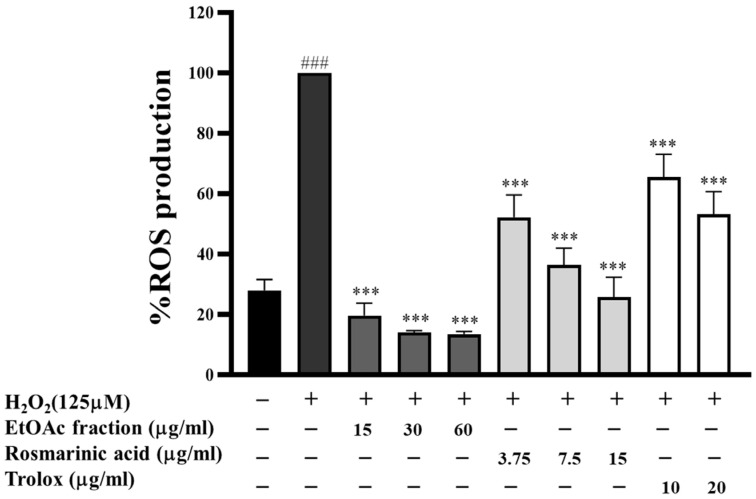
Effect of the EtOAc fraction and rosmarinic acid on H_2_O_2_-induced ROS in HepG2 cells. Trolox was used as a positive control. All assays were performed in triplicate; data are presented as mean ± standard deviation (^###^ *p* < 0.001 vs. the control group; *** *p* < 0.001 vs. (+) H_2_O_2_-induced group).

To assess the mitigating effect against oxidative stress, the influence of the EtOAc fraction, rosmarinic acid, and trolox on intracellular ROS content in HepG2 cells was established. The DCFH-DA assay, which is frequently employed in the assessment of oxidative stress, was utilized to quantify the production of ROS within the cells. As shown in Figure 3, H_2_O_2_ (125 µM) increased the level of ROS generation approximately 4-fold (*p* < 0.001) compared to the untreated cells. The EtOAc fraction (15–60 µg/mL) dramatically decreased ROS generation in a dose-dependent manner. It demonstrated a greater potential to reduce ROS generation than standard rosmarinic acid and trolox at the same concentration. Our findings indicate that the EtOAc fraction scavenged extreme intracellular ROS induced by H_2_O_2_ in HepG2 cells more effectively than the traditional antioxidants trolox and standard rosmarinic acid.

### 3.5. Inhibitory Effect of EtOAc Fraction on Lipid Peroxidation

**Figure 4:**
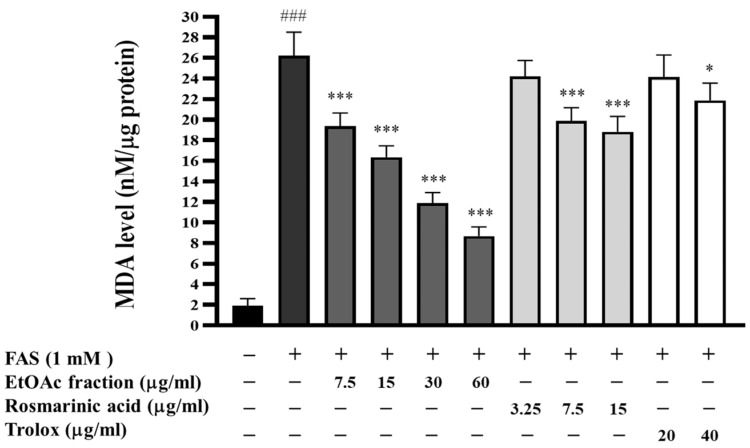
Effect of the EtOAc fraction and rosmarinic acid on lipid peroxidation in HepG2 cells. Trolox was used as a positive control. All assays were performed in triplicate; data are presented as mean ± standard deviation (^###^ *p* < 0.001 vs. the control group; * *p* < 0.05, *** *p* < 0.001 vs. (+) FAS-induced group).

Intracellular ROS production can cause lipid peroxidation and the degradation of lipid bilayer membranes by reacting with the polyunsaturated fatty acids (PUFAs) of lipid membranes. The amount of the lipid peroxidation end product was measured using the thiobarbituric acid reactive substances (TBARs) assay. As shown in Figure 4, treating HepG2 cells with ferrous ammonium sulfate (FAS) (1 mM) resulted in a significant, approximately 13-fold (*p* < 0.001), increase in the MDA level, compared to the untreated cells. Conversely, the MDA level in the FAS-treated cells was dramatically reduced upon treatment with the EtOAc fraction (7.5–60 μg/mL), rosmarinic acid (7.5–15 μg/mL), and trolox (40 μg/mL). According to our findings, it is indicated that the EtOAc fraction can scavenge ROS in H_2_O_2_-induced HepG2 cells and shows an ability to prevent lipid peroxidation in these cells. Furthermore, the antioxidant compounds rosmarinic acid and trolox were found to be less effective than the EtOAc fraction in reducing lipid peroxidation in HepG2 cells, as well as inhibiting the formation of intracellular ROS in prior experiments.

**Figure 5:**
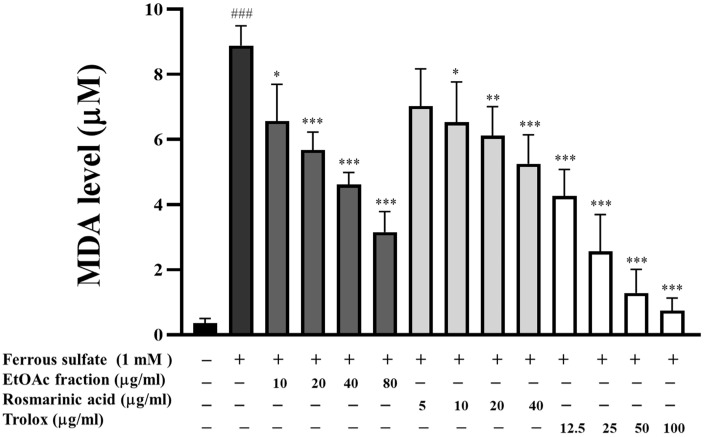
Effect of the EtOAc fraction and rosmarinic acid on lipid peroxidation in egg yolk. Trolox was used as a positive control. All assays were performed in triplicate; data are presented as mean ± standard deviation (^###^ *p* < 0.001 vs. the control group; * *p* < 0.05, ** *p* < 0.01, *** *p* < 0.001 vs. (+) ferrous sulfate-induced group).

In the in vitro TBARS assay, performed in egg yolk homogenates, ferrous sulfate-induced lipid peroxidation resulted in a dramatic, approximately 9-fold (*p* < 0.001), increase in the MDA level, compared to the control group (Figure 5). On the other hand, the EtOAc fraction inhibited the lipid peroxidation induced by ferrous sulfate in egg yolk homogenate in a concentration-dependent manner. It exhibited great potential compared to the standard rosmarinic acid and trolox.

### 3.6. Effect of EtOAc Fraction on LPS-Induced Nitric Oxide Production in RAW 264.7 Cells

**Figure 6:**
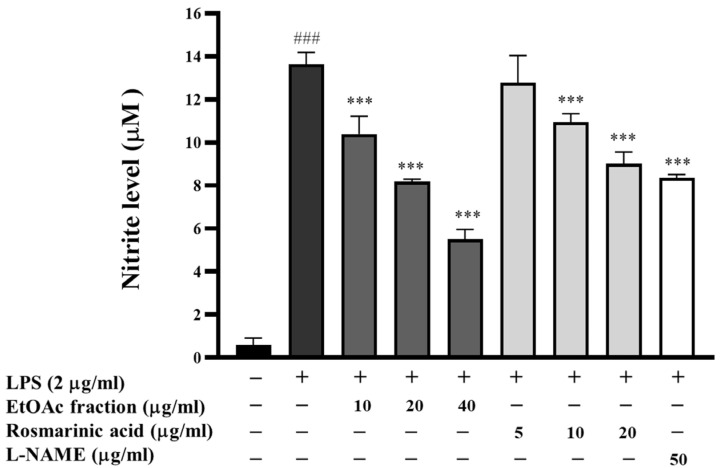
Effect of the EtOAc fraction and rosmarinic acid on nitric oxide production. L-NAME was used as a positive control. All assays were performed in triplicate; data are presented as mean ± standard deviation (^###^ *p* < 0.001 vs. the control group; *** *p* < 0.001 vs. (+) LPS-induced group).

Nitric oxide (NO) is highly unstable in biological systems and quickly oxidizes to nitrite (NO_2_^−^), so nitrite measurement is frequently used to indicate NO production. The Griess reaction was used to determine the quantity of nitrite released into the culture medium, the level of nitrite contained in the culture supernatants was defined as an indirect indicator of nitric oxide generation, and the concentration of nitrite was measured using sodium nitrite as a standard. In this study, the effect of the EtOAc fraction on NO generation in LPS-induced RAW 267.4 cells was determined using the Griess assay. As shown in Figure 6, the NO level was dramatically increased by 14-fold (*p* < 0.001) after LPS treatment. On the other hand, the NO generation in RAW 264.7 cells stimulated with LPS was greatly reduced by treatment with EtOAc fractions in a dose-dependent manner (1.31–2.48-fold). The positive controls, rosmarinic acid and L-NAME, also resulted in 1.07–1.51 and 1.63-fold decreases in the amount of NO generated, respectively. It can be concluded that the EtOAc fraction was more effective than rosmarinic acid and L-NAME in preventing NO formation. According to our study, the EtOAc fraction effectively reduced the generation of NO, suggesting that it could be helpful in suppressing the NO production process.

### 3.7. Effect of EtOAc Fraction on Iron Chelation Activity

**Figure 7:**
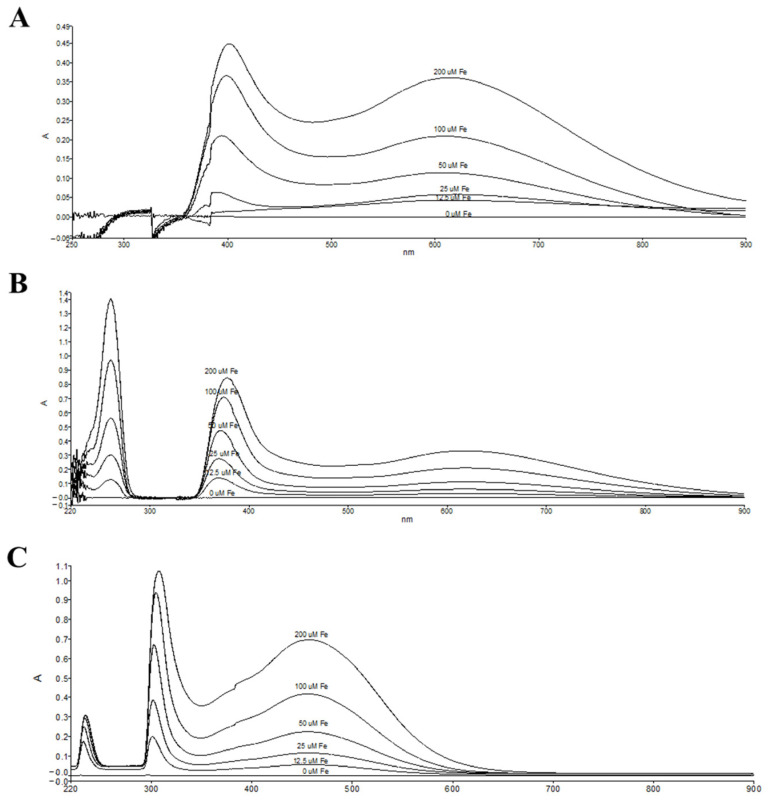
Iron-binding properties of Fe-NTA (0–200 μM) and 500 μg/mL EtOAc fraction (**A**), 100 μg/mL rosmarinic acid (**B**), or 14 μg/mL deferiprone (**C**). Iron complexes were measured by UV–VIS spectrophotometer at 220–900 nm.

Iron can cause the generation of free radicals via the Fenton and Haber–Weiss reactions. The iron chelation activity of the EtOAc fraction was assessed using a ferric nitrilotriacetate (Fe^3+^-NTA)-binding assay, and the absorption intensity of the complex was measured with a spectrophotometer. This chelation assay was conducted to evaluate the reduction in reactive oxygen species formation by chelating metal ions, which demonstrated the iron-binding activity. The EtOAc fraction exhibited iron-chelating properties by binding ferric ions to form complexes, and the predominant peak of the ferric ion and EtOAc fraction complexes were shown at 400 and 600 nm, respectively (Figure 7A). The increase in ferric ion concentration (12.5–200 μM) results in a dose-dependent enhancement of complex absorbance. Similarly, rosmarinic acid shows iron-chelating properties in a dose-dependent manner (12.5–200 μM). It exhibits the predominant absorption complexes at 260, 376, and 610 nm, respectively (Figure 7B). Compared with a standard iron chelator, deferiprone, the results exhibit a ferric ion-binding property to form an orange-yellow complex solution and show predominant peaks at 220, 300, and 450 nm (Figure 7C). These results indicated that the EtOAc fraction displays an iron-chelating activity, suggesting it would reduce iron-induced oxidative stress and overload conditions.

### 3.8. Effect of EtOAc Fraction on Iron Chelation in HepG2 Cells

**Figure 8:**
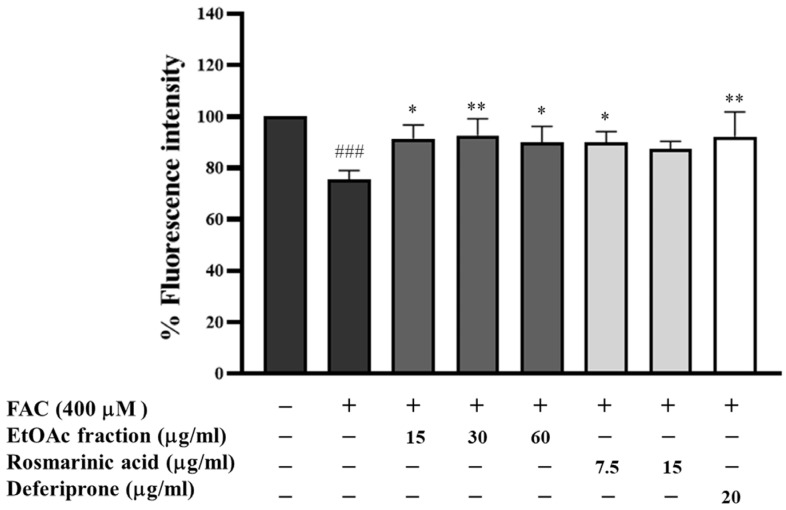
The fluorescence intensity represents LIP in hepatocytes. Iron-loaded HepG2 cells were treated with the EtOAc fraction (15–60 μg/mL), rosmarinic acid (7.5 and 15 μg/mL), and a standard iron chelator deferiprone (20 μg/mL) for 24 h. All assays were performed in triplicate; data are presented as mean ± standard deviation (^###^ *p* < 0.001 vs. the control group, * *p* < 0.05, ** *p* < 0.01 vs. iron-loaded group (400 μM FAC)).

Excessive iron can accumulate in the liver, forming a labile iron pool (LIP) and generating ROS, which can lead to tissue damage. Therefore, LIP is the target to examine the iron-chelating properties in cells. The effect of the EtOAc fraction on intracellular iron chelation was studied in HepG2 cells. Iron-loaded HepG2 cells were induced with ferric ammonium citrate (FAC) for 24 h and then treated with the EtOAc fraction, rosmarinic acid, or deferiprone (a standard iron chelator) for 24 h. The LIP level represents the inverse of the percentage of fluorescence intensity (% FI). HepG2 cells incubated with 400 μM FAC alone showed a significant increase in LIP levels (*p* < 0.001) compared to non-treated cells (Figure 8). Conversely, post-treatment with the EtOAc fraction (15–60 μg/mL), rosmarinic acid (7.5 μg/mL), or deferiprone (20 μg/mL) resulted in a significant decrease in LIP levels compared to the iron-loaded HepG2 cells. Rosmarinic acid at a 15 μg/mL concentration slightly decreased LIP levels in iron-loaded HepG2 cells. These results indicated that the EtOAc fraction shows an intracellular iron-chelating activity.

### 3.9. Effect of EtOAc Fraction on Transferrin Receptor (TfR) Expression in HepG2 Cells

**Figure 9:**
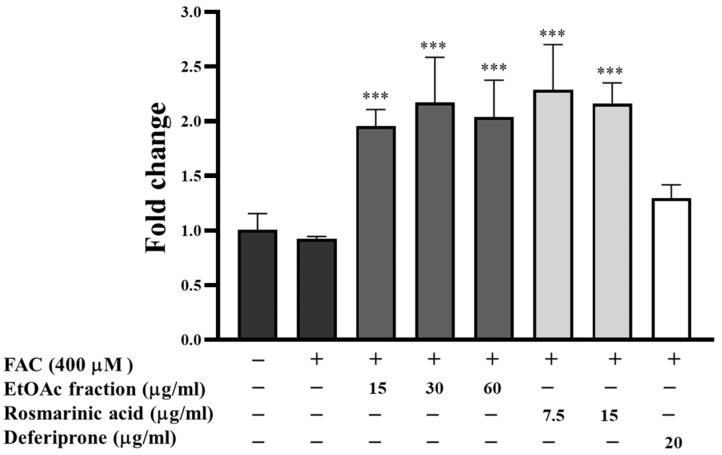
The expression of the *TfR* gene in hepatocytes. Iron-loaded HepG2 cells were treated with the EtOAc fraction (15–60 μg/mL), rosmarinic acid (7.5 and 15 μg/mL), and a standard iron chelator deferiprone (20 μg/mL) for 24 h. All assays were performed in triplicate; data are presented as mean ± standard deviation (*** *p* < 0.001 vs. (+) iron-loaded group (400 μM FAC)).

Systemic iron levels control iron uptake into the cell. Intracellular iron deprivation activates transferrin receptor (TfR) expression on cell membranes for iron uptake. The expression of the *TfR* gene in iron-loaded HepG2 cells was evaluated using real-time RT-PCR. The results indicated that TfR expression was slightly decreased in iron-loaded HepG2 cells (400 μM FAC) compared to the control group (Figure 9). Interestingly, the EtOAc fraction (15–60 μg/mL) and rosmarinic acid (7.5 and 15 μg/mL) significantly upregulated TfR expression compared to the iron-loaded group (*p* < 0.001). Deferiprone slightly induced TfR expression, compared to the iron-loaded group. These results suggest that the EtOAc fraction and rosmarinic acid could reduce intracellular iron levels in iron-loaded HepG2 cells, leading to an increased expression of TfR.

## 4. Discussion

*Perilla frutescens* extracts are rich in phytochemicals that exhibit antioxidant activity, mainly associated with phenolic compounds [30,31]. From our previous studies, the primary components of the leaf, seed, and seed residue of the *Perilla* extract were phenolic acids and flavonoids, predominantly rosmarinic acid (RA) [21,32,33]. It is widely recognized that these secondary metabolites have antioxidant, anti-inflammation, and anticancer properties [18,34,35,36]. Table 1 shows that phenolics, flavonoids, and RA were present in higher amounts in the EtOAc fraction than in the ethanolic extract. The results corroborate previous reports that RA was mainly predominant in the EtOAc fraction, and this fraction also had the highest phenolic and flavonoid content [37,38,39]. It has been reported that ethyl acetate was used for the purification of rosmarinic acid in the final step of the solvent extraction method [40].

The antioxidative properties of natural compounds are characterized through various distinct mechanisms, such as scavenging free radicals and chelating metal ions like iron [41]. In this study, we investigated the free radical scavenging capacities of the EtOAc fraction, using DPPH and ABTS, and their ferric reducing capacities using the FRAP assay. The DPPH and ABTS assays measured the capability to donate hydrogen atoms or electrons from antioxidant molecules to DPPH^•^/ABTS^•+^ [42], while the FRAP assay was used to measure the reduction of ferric ion (Fe^3+^) to ferrous ion (Fe^2+^) [43]. Our results demonstrated that DPPH and ABTS free radical scavenging activities were more effectively inhibited by the EtOAc fraction in vitro. Reducing power indicates the potential effectiveness of an antioxidant compound [44,45]. The results of the FRAP assay indicated that the EtOAc fraction exhibited the highest reducing power. In addition, the iron-chelating properties of the EtOAc fraction were effective in binding the ferric ion (ferric-NTA) in a concentration-dependent manner, and the complexes showed absorbance at 400 and 600 nm. Similar to previous studies, the EtOAc fraction showed strong antioxidant activities in scavenging free radicals and reducing power capacities through iron binding [37,38]. Therefore, for the partial partitioning of RA-enriched extract from PS with strong antioxidant and iron-binding capabilities, ethyl acetate may be a suitable solvent in vitro. Excessive iron can generate reactive oxygen species (ROS), especially hydroxyl radicals, via the Fenton reaction [46]. Recently, interest has been shown in researching phytochelators’ ability to alleviate iron toxicity. Their molecular structure presents iron-binding sites, such as carbonyl (CO), hydroxyl (OH), amino (NH_2_), and sulfhydryl (SH) groups, which possess electron donor atoms that enable metal complex formation [47]. The complexation of iron with phytochelator can inhibit hydroxyl radicals’ generation and result in potent antioxidant activity [48]. The major compound of the EtOAc fraction is rosmarinic acid, which exhibits iron-binding properties. The chemical structure of RA presents with a carboxylic acid (COOH), a hydroxyl (OH), and aromatic hydroxyl groups; these functional groups might have iron-binding capacities. In addition, RA shows ferrous ions (Fe^2+^)-binding activities to reduce hydroxyl radical production [49]. ROS can attack vital macromolecules such as lipids, proteins, and DNA in the cell, leading to damage [50,51]. As hepatocytes are the primary site of iron accumulation, excessive iron can accumulate in the liver, resulting in the production of ROS. Therefore, RA-enriched extract from PS was assessed for intracellular ROS scavenging activity in HepG2 cells for evaluating its protective cellular damage effect in vitro. The results of the intracellular ROS scavenging activity suggested that the EtOAc fraction at non-toxic doses significantly inhibited intracellular ROS generation in H_2_O_2_-activated HepG 2 cells, and these were greater than those of rosmarinic acid and trolox in the same inhibitory activity. In addition, lipid peroxidation was a product of the attacking cell membrane via ROS [52]. The lipid peroxidation products, such as malondialdehyde (MDA), can oxidize thiols in proteins and cause protein dysfunction, which causes the development of disorders including inflammation, cancer, and cardiovascular and neurodegenerative disease [53,54]. In this study, we investigated the inhibitory effect of the EtOAc fraction on lipid peroxidation in HepG2 cells and egg yolk. The results revealed that the EtOAc fraction at non-toxic doses significantly decreased lipid peroxidation in HepG2 cell and egg yolk models.

Reactive nitrogen species (RNS), including nitric oxide (NO) and peroxynitrite, are a subclass of ROS. The overproduction of NO affects protein structure and function, leading to physiologic dysfunctions [55,56,57]. Thus, the inhibitory effect of the EtOAc fraction on nitric oxide production in RAW264.7 cells was investigated. Interestingly, the EtOAc fraction markedly reduced NO production in LPS-induced RAW264.7 cells, as well as RA, which was the major component of the EtOAc fraction. Consequently, our data were related to previous reports of *Perilla* extract inhibiting NO production in cell cultures [37,58,59]. These findings lead us to consider that RA-enriched extract from PS may be crucial in protecting against intracellular ROS/RNS and their products.

To evaluate the iron-chelating properties of the EtOAc fraction, we examined the labile iron pool (LIP) level in HepG2 cells. As previously discussed, excessive iron can penetrate and accumulate in the liver, forming the LIP, which can subsequently stimulate the production of ROS [60]. Consequently, it represents a target for iron chelators, which can diminish intracellular iron toxicity and increase cellular protection [61]. Our findings showed that the EtOAc fraction significantly reduced the LIP in iron-loaded HepG2 cells. Additionally, we examined the expression of the transferrin receptor (TfR), which is crucial for cellular iron uptake and maintaining iron homeostasis [62]. Low iron levels in cells increase TfR expression, but high iron levels decrease TfR expression on cell membranes [61]. The results revealed that the EtOAc fraction significantly induced TfR expression in iron-loaded HepG2 cells. It is plausible to conclude that the EtOAc fraction has the potency of an iron chelator agent against iron loading in HepG2 cells.

## 5. Conclusions

This work is an attempt to identify the biological potential of the *Perilla frutescens* seed in terms of its iron-chelating property. The EtOAc fraction obtained from crude ethanolic extract of *Perilla* seeds was evaluated for antioxidant, hepatoprotective, and iron-chelating activities. The EtOAc fraction showed a great ability to suppress cellular ROS production and prevent lipid peroxidation, suggesting its potential to protect against iron-induced oxidative damage to liver cells. Moreover, the EtOAc fraction exhibited potent iron-chelating activities in iron-loaded HepG2 cells. Consequently, the EtOAc fraction of *Perilla frutescens* is essential for establishing its safety and toxicity profiles through comprehensive preclinical studies. Thus, future research should focus on determining the potential adverse effects, optimal dosage, and long-term safety of this extract in animal models. Preclinical safety and efficacy testing will provide the critical information necessary for progressing its development to human clinical trials.

## Figures and Tables

**Table 1:** Percent yield, TPC, TFC, RAC, and antioxidant activities in PS extract fractions.

Samples	%Yield	TPC	TFC	RAC	IC_50_ (µg/mL)		FRAP Value
					DPPH Assay	ABTS Assay	
EtOH	18.68	180.56 ± 4.47	72.27 ± 3.58	41.77 ± 0.02	100.75 ± 3.91	35.92 ± 0.70	486.23 ± 27.22
Hex	5.79	80.60 ± 6.62	17.96 ± 0.73	4.74 ± 0.20	244.66 ± 3.38	100.97 ± 2.41	169.44 ± 13.38
DCM	2.69	192.70 ± 5.65	35.26 ± 1.35	10.42 ± 0.07	130.35 ± 4.91	24.32 ± 0.50	341.16 ± 14.95
EtOAc	2.24	1314.05 ± 23.04	329.30 ± 10.37	393.81 ± 0.05	13.35 ± 0.81	3.98 ± 0.13	5062.95 ± 354.87
Water	70.40	136.12 ± 1.69	62.66 ± 2.47	32.92 ± 0.01	123.77 ± 3.19	31.38 ± 0.79	403.91 ± 24.93
Rosmarinic acid	-	-	-	-	8.69 ± 0.37	5.45 ± 0.04	7775.37 ± 439.56
Vitamin C	-	-	-	-	5.47 ± 0.11	5.18 ± 0.06	4347.10 ± 57.09
Trolox	-	-	-	-	5.20 ± 0.02	5.17 ± 0.04	4885.00 ± 95.35

%Yield; TPC, total phenolic content (mg GAE/g extract); TFC, total flavonoid content (mg CE/g extract); RAC, rosmarinic acid content (mg/g extract); FRAP value (mg Fe^2+^ equivalent/g extract).

## Data Availability

Data are contained within the article.
